# Postoperative MRI Findings Following Conventional and Extralevator Abdominoperineal Excision in Low Rectal Cancer

**DOI:** 10.3389/fsurg.2021.771107

**Published:** 2021-11-16

**Authors:** Kim Morgenstjerne Oerskov, Peter Bondeven, Søren Laurberg, Rikke H. Hagemann-Madsen, Henrik Kidmose Christensen, Henrik Lauridsen, Bodil Ginnerup Pedersen

**Affiliations:** ^1^Department of Radiology, Aarhus University Hospital, Aarhus, Denmark; ^2^Department of Surgery, Randers Regional Hospital, Randers, Denmark; ^3^Department of Surgery, Aarhus University Hospital, Aarhus, Denmark; ^4^Department of Clinical Medicine, Aarhus University, Aarhus, Denmark; ^5^Department of Pathology, Lillebaelt Hospital, Vejle, Denmark

**Keywords:** rectal cancer, magnetic resonance imaging, extralevator abdominoperineal excision, ELAPE, APE

## Abstract

**Aim:** The disparity in outcomes for low rectal cancer may reflect differences in operative approach and quality. The extralevator abdominoperineal excision (ELAPE) was developed to reduce margin involvement in low rectal cancers by widening the excision of the conventional abdominoperineal excision (c-APE) to include the posterior pelvic diaphragm. This study aimed to determine the prevalence and localization of inadvertent residual pelvic diaphragm on postoperative MRI after intended ELAPE and c-APE.

**Methods:** A total of 147 patients treated with c-APE or ELAPE for rectal cancer were included. Postoperative MRI was performed on 51% of the cohort (*n* = 75) and evaluated with regard to the residual pelvic diaphragm by a radiologist trained in pelvic MRI. Patient records, histopathological reports, and standardized photographs were assessed. Pathology and MRI findings were evaluated independently in a blinded fashion. Additionally, preoperative MRIs were evaluated for possible risk factors for margin involvement.

**Results:** Magnetic resonance imaging-detected residual pelvic diaphragm was identified in 45 (75.4%) of 61 patients who underwent ELAPE and in 14 (100%) of 14 patients who underwent c-APE. An increased risk of margin involvement was observed in anteriorly oriented tumors with 16 (22%) of 73 anteriorly oriented tumors presenting with margin involvement vs. 7 (9%) of 74 non-anteriorly oriented tumors (*p* = 0.038).

**Conclusion:** Residual pelvic diaphragm following abdominoperineal excision can be depicted by postoperative MRI. Inadvertent residual pelvic diaphragm (RPD) was commonly found in the series of patients treated with the ELAPE technique. Anterior tumor orientation was a risk factor for circumferential resection margin (CRM) involvement regardless of surgical approach.

## Introduction

Treatment of patients with rectal cancer has improved dramatically with the adoption of mesorectal excision surgery (1–4), the introduction of MRI for preoperative tumor staging (5–7), multidisciplinary team (MDT) conferences for the planning of treatment (8), and use of preoperative chemoradiotherapy (CRT). However, outcomes for patients with locally advanced low rectal cancers, which necessitate abdominoperineal excision (APE), have been inferior to those following sphincter-preserving surgery for mid- or upper rectal cancer with poorer survival and a higher risk of local recurrence (9–11). The observed inferior outcomes in low rectal cancer are most likely multifactorial, including high rates of positive circumferential resection margin (CRM) involvement and specimen perforation. These outcomes may in part be explained on the basis of the surgical planes during resection when conventional APE (c-APE) is performed (4, 6, 7, 9–11). This aspect of APE specimens was first identified in 2002 (9) and subsequently verified in a joint study of APE specimens in the Dutch total mesorectal excision (TME) trial (10).

To reduce margin involvement and specimen perforation, the “extralevator APE” (ELAPE) was promoted by Holm et al. (12). The ELAPE involves removing the levators attached to the lower mesorectum and the entire anal canal with internal and external sphincters and a greater or lesser volume of ischioanal fat. The procedure is performed under direct vision, leaving only the most anterior parts of the levator ani *in situ*, and may provide the critical extra margin of protection around a locally advanced low rectal tumor.

In 2008, West et al. compared ELAPE specimens to c-APE specimens and demonstrated markedly reduced rates of CRM involvement and specimen perforation. Consistent with this finding, an increased amount of tissue was removed by the ELAPE technique compared with the c-APE technique (13). Standardization and quality assurance by training and pathological audit were implemented in the major trials to ensure that optimal surgery was performed (4, 14, 15). Thus, data on the problems of c-APE seemed abundant, and evidence of the superiority of the ELAPE was accumulating.

However, in 2014, Ortiz et al. published a multicenter study comparing ELAPE to c-APE, finding that ELAPE did not improve the rates of involved CRM, tumor perforation, local recurrence rates, or mortality (16). Others have proclaimed that ELAPE should not be the surgery of choice for low rectal cancers, although advocating that the selective use of the procedure might be warranted (17–21). However, patients may more often suffer from wound complications and perineal pain after ELAPE than after c-APE (22–24).

Thus, opinions range from authors advocating the widespread implementation of ELAPE for the treatment of low rectal cancers to the proposition that the procedure be filed under “nunquam iterum” —never again (25).

The disparity in outcomes may reflect differences in operative approach and quality. In 2018, Holm argued that since no formal standardization of the c-APE exists, the procedure has gradually taken on characteristics of the ELAPE, thus explaining why rates of involved CRM and local recurrence in c-APE have improved (26). Using postoperative MRI allows the assessment of the extent and completeness of mesorectal excision after surgery for rectal cancer (27, 28). This makes postoperative MRI an expedient method for quality assessment of both surgery and pathological assessment of the specimen.

In this study, we aimed to investigate the prevalence and localization of inadvertent residual pelvic diaphragm (RPD) on postoperative MRI after ELAPE and c-APE. Clinical data were analyzed for potential risk factors for having RPD at postoperative MRI, and for the involvement of the CRM at pathological evaluation.

## Methods

In 2007, an audit on the quality of rectal cancer treatment and surgery was implemented at Aarhus University Hospital, Denmark. The audit was part of a large regional audit with a focus on postgraduate training of colorectal MDTs in the North and Central Denmark Region. This study was approved as a quality assurance project by the local ethics committee with no need for oral or written consent required by Danish law.

### Population

**Figure 1:**
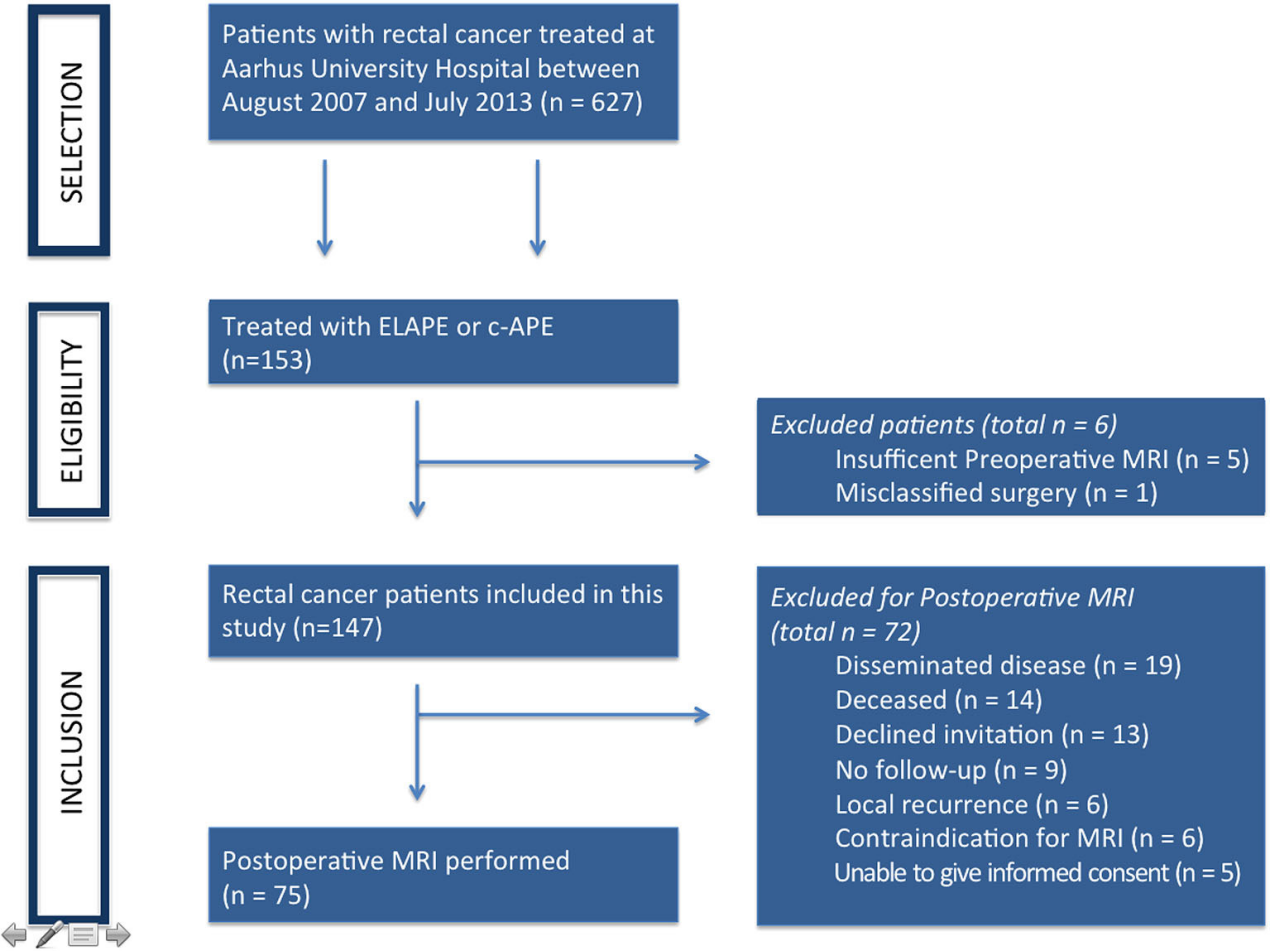
Patient inclusion flowchart.

The Department of Surgery at Aarhus University Hospital had a primary catchment population of 400.000 inhabitants during the study period, during which approx. one hundred and twenty patients with rectal cancer were treated annually. The department serves as a secondary referral center for advanced low rectal cancer in the region (population 1.25 million) and as the tertiary referral center for very advanced as well as locally recurrent rectal cancer in Denmark (population 5.8 million). Patients with low rectal adenocarcinoma who underwent ELAPE or c-APE between October 2007 and July 2013 were included (Figure 1). Consecutive patients were invited for postoperative MRI of the pelvis. Excluded were patients with disseminated disease, previous diagnosis of local recurrence, contraindication for MRI, unable to give informed consent, or deceased.

A total of 147 patients treated with ELAPE or c-APE between 2007 and 2013 were included. Patient and treatment characteristics are shown in Table 1. Of the 147 patients, 75 (51%) had postoperative MRI performed. Postoperative MRI was performed a median of 12 months after primary surgery.

**Table 1:** Demographic and clinical data.

	***N* = 147**
Sex ratio, M:F	91:56 (38.1% F)
Median age in years (range)	67 (40–89)
Distance of primary tumor to anal verge by rigid proctoscopy in centimeters	
→ 0–1.9	10 (6.8)
→ 2–3.9	54 (36.7)
→ 4–5.9	42 (28.6)
→ ≥6	12 (8.2)
→ Missing	29 (19.7)
Neoadjuvant therapy	
→ None	54 (36.7)
→ CRT	93 (63.3)
T-category on MRI	
→ T2	36 (24.5)
→ T3	78 (53.1)
→ T4	32 (21.7)
→ Tx	1 (0.7)
Tumor orientation on MRI	
→ Anterior	73 (49.3)
→ Other	74 (50.7)
Surgery	
→ ELAPE	125 (85.0)
→ c-APE	22 (15.0)
Pathological T-category^*^	
→ pT0	10 (6.8)
→ pT1	5 (3.4)
→ pT2	48 (32.7)
→ pT3	71 (48.3)
→ pT4	13 (8.8)
Circumferential resection margin	
→ Not involved	124 (84.4)
→ Involved	23 (15.6)
Venous invasion	
→ V0	106 (72.1)
→ V1-V2	41 (27.9)
Lymph node involvement	
→ N0	113 (76.9)
→ N1-N2	33 (22.4)
→ Missing	1 (0.7)
Mesorectal plane of surgery	
→ Mesorectal	39 (26.5)
→ Intramesorectal	43 (29.3)
→ Musc. Propria	64 (43.5)
→ Not reported	1 (0.7)
Perineal plane of surgery	
→ Extralevator plane	19 (12.9)
→ Sphincteric plane	59 (40.1)
→ Intramuscular/submucosal plane	60 (40.8)
→ Not reported	9 (6.2)
Local recurrence	
→ Yes	11 (7.5)
→ No	136 (92.5)

*Values in parentheses are percentages unless indicated otherwise*.

* *Based on pathological evaluation of excised specimen (the pathological tumor category for the 94 patients who had preoperative adjuvant therapy (ypT) was: T0, 10; T1, 4; T2, 24; T3, 43; T4, 11)*.

*CRM, circumferential resection margin; MRI, magnetic resonance imaging*.

Data on patient characteristics and clinical information were obtained from clinical records. Throughout the study period, the preferred and standard surgical approach for low rectal cancer at Aarhus University Hospital was the ELAPE performed with the intent of removing all posterior muscular pelvic diaphragm (29). Professor Holm introduced and supervised the procedure at the hospital while appointed there.

Low rectal cancer was defined as tumors located between 0 and 5 cm from the anal verge, measured by rigid proctoscopy. Topographical relations of the tumor were weighted over standardized measurements, and thus, selected patients with tumors above 5 cm from the anal verge but within a short distance of the levators at preoperative MRI were treated with ELAPE or c-APE and consequently included in this study.

In accordance with Danish guidelines, patients with low rectal and UICC TNM category T3 or T4 tumors were referred for long-course neoadjuvant CRT. Treatment planning including the decision of surgical approach was made at a multidisciplinary conference.

### Postoperative MRI

A dedicated MRI protocol was developed, including sagittal, axial, and coronal T2-weighted turbo spin echo images, slice thickness of 4 mm, in addition to a sagittal, short T1 inversion recovery (STIR) sequence of the bony pelvis and a sagittal T2 3D sequence of the pelvis. Postoperative MRIs were performed a minimum of 6 months after surgery to avoid confusion with postoperative changes.

### Tumor Location and Orientation in the Axial Plane

**Figure 2:**
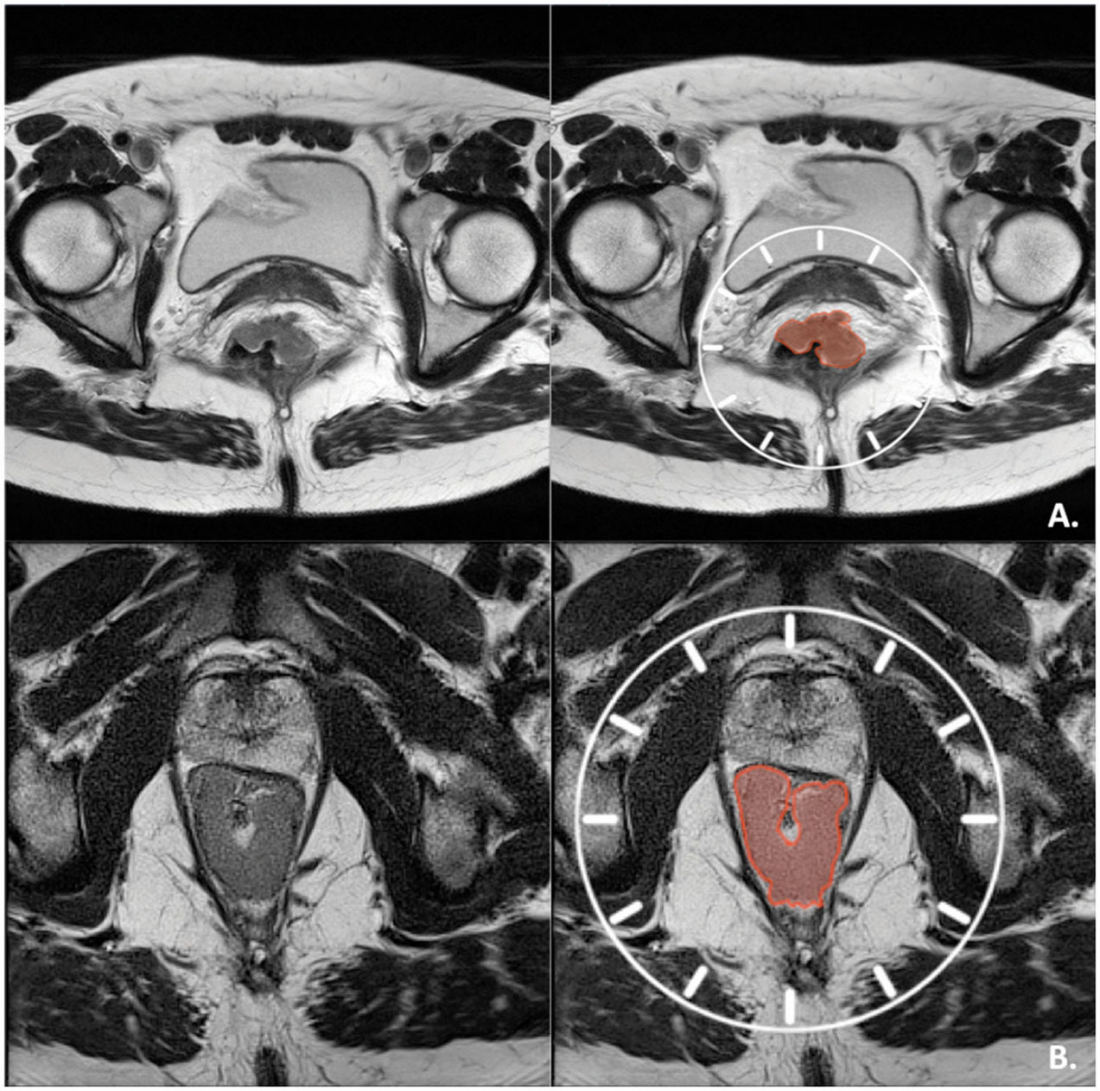
Tumor orientation. **(A)** Axial T2-weighted view of an anterior mrT3 tumor with center and invasive component between 11 and 1 o'clock. **(B)** Axial T2-weighted view of a non-anterior, early mrT3 tumor with center and invasive component between 5 and 7 o'clock.

Tumor center and location(s) of invasive growth (if applicable) were determined on the preoperative MRIs. Tumors with a center or invasive growth between 10 o'clock and 2 o'clock were classified as “anterior,” while tumors with a center or invasive growth between 2 and 10 o'clock were classified as “non-anterior” (Figure 2).

### Residual Pelvic Diaphragm

**Figure 3:**
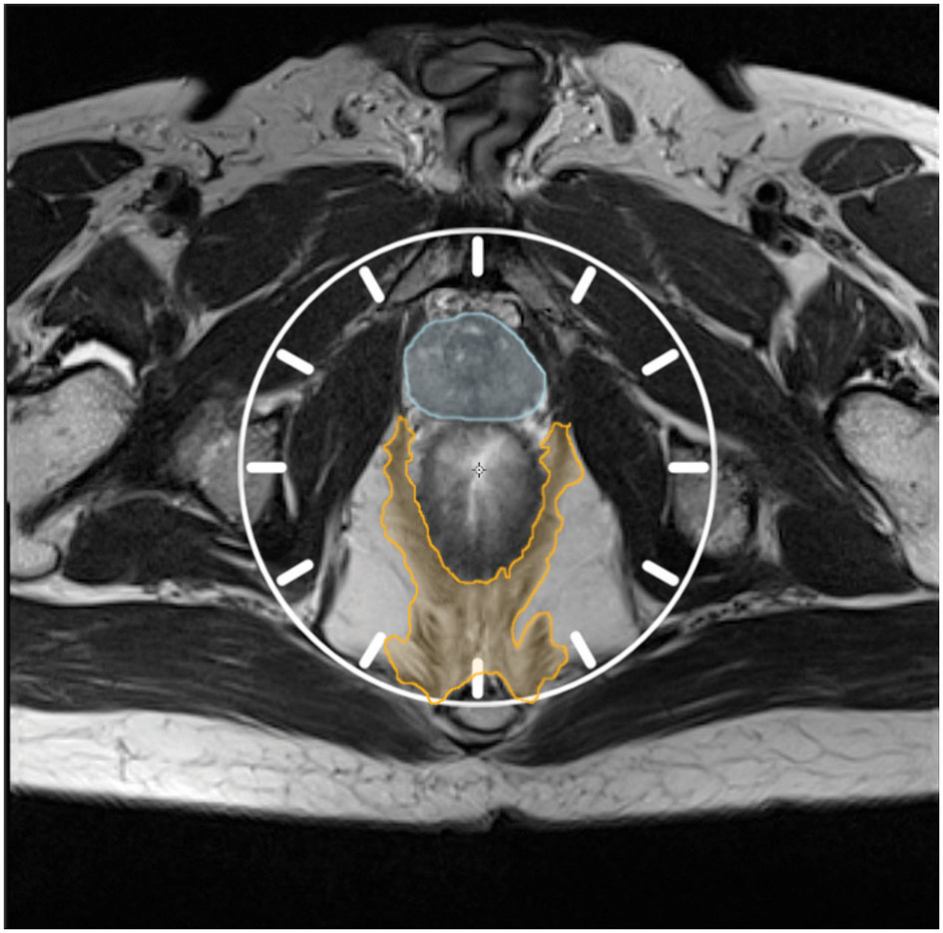
Visualizing the axial plane of T2w MRI as the face of a clock. Orange hatching: Pelvic diaphragm. Blue hatching: Prostate.

The residual pelvic diaphragm was defined as any remaining levator ani and/or coccygeal muscle visible on postoperative MRI in the two posterior quadrants of the pelvis [from 3 to 6 and 6 to 9 o'clock if visualizing the axial plane of an MRI of the mesorectum and pelvic diaphragm as the face of a clock (Figure 3)]. The MRI examinations were all evaluated by a dedicated multidisciplinary team radiologist subspecialized in pelvic MRI and reviewed together with co-author PB for consensus. The multidisciplinary team radiologist was blinded to all clinical data with the exception of the preoperative MRI examination.

### Pathology

The pathological evaluation followed a standardized protocol with the assessment of the surgical plane achieved in the mesorectal and the perineal segments (30, 31). The CRM was considered involved if a distance of 1 mm or less was observed between any vital tumor cell and the resection margin. Inspecting standardized photo documentation, an experienced colorectal pathologist retrospectively evaluated the specimens for surgical plane and volume defects in the pelvic diaphragm. The pathological assessment was blinded to the clinical data and MRI findings.

### 3D Rendering of MRI

Three-dimensional renders of the pelvic diaphragm at T2w images were made to ensure a better spatial understanding of the anatomy of the pelvic diaphragm and for aiding visualization of the surgical planes before and after ELAPE or c-APE surgery (32). Amira version 5.6 (Thermo Fischer Scientific, Waltham, MA, United States) at a Windows platform was used for image segmentation and 3D rendering. Segmentation was done semi-automatically and reviewed by an experienced radiologist with more than 10 years of experience with pelvic MRI.

### Statistical Analysis

For comparison of categorical data distributions, χ2-test, Fisher's exact test, or the Fisher–Freeman–Halton test was used. *P* < 0.05 were considered statistically significant. The programming language “R” (R Foundation for Statistical Computing, Vienna, Austria) and the coding program “RStudio” (RStudio, Inc., Boston, MA) were used for statistical analysis.

## Results

### Detection of RPD on Postoperative MRI

**Figure 4:**
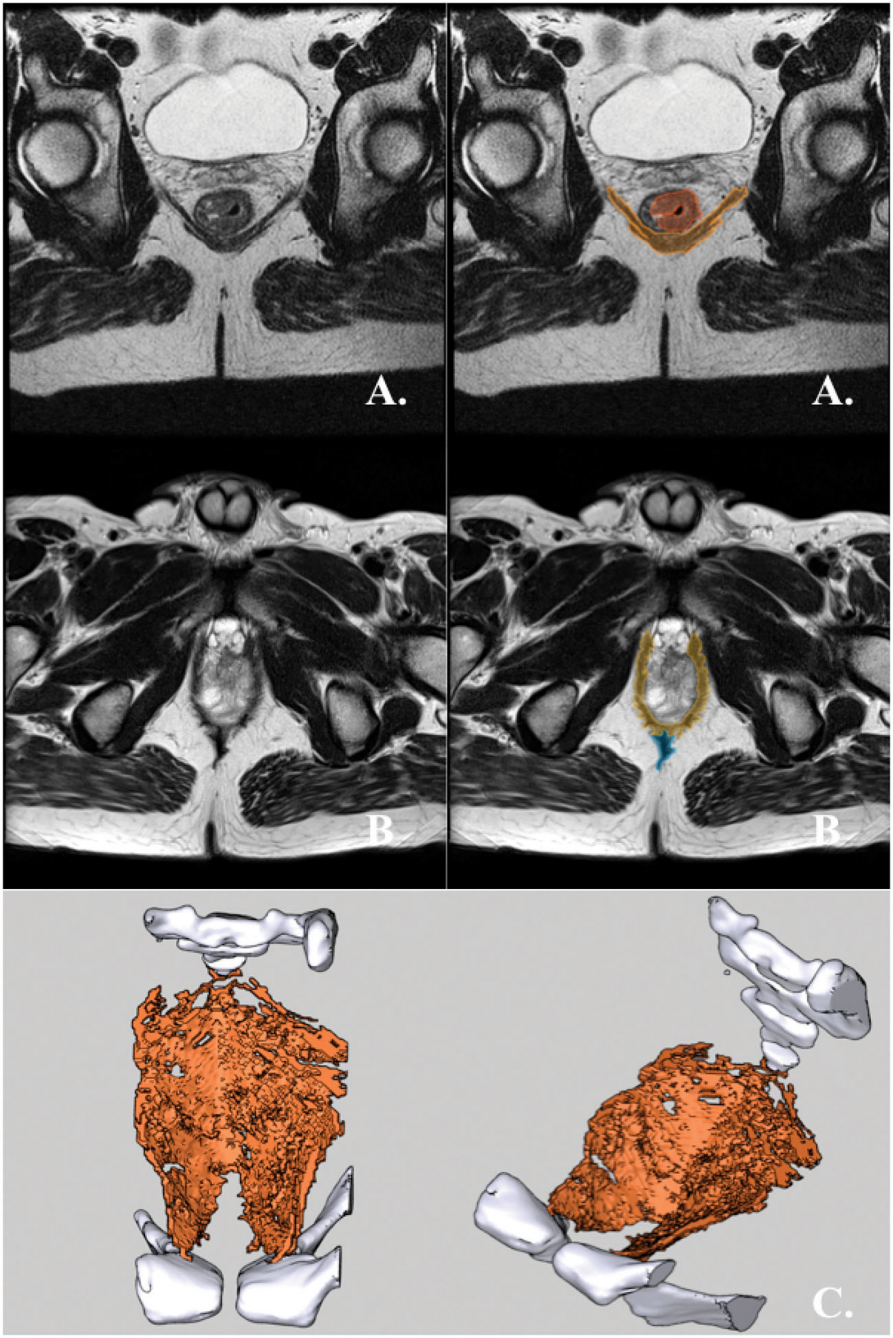
c-APE. **(A)** Preoperative axial T2-weighted MRI of a rectal cancer patient with an mrT3-tumor with invasive growth between 2 and 4 o'clock. RPD is hatched in orange. The tumor is hatched in red. **(B)** Postoperative axial T2-weighted MRI of the same patient after cAPE. RPD is hatched in orange. Cicatrice is hatched in blue. **(C)** The RPD of the same patient, 3D-rendered and shown in superior and antero-lateral superior views. The rendering is made from the above postoperative MRI. The bony pelvis is rendered in white, while RPD is rendered in orange. No bio-mesh was used for supporting the closure of the defect in the pelvic diaphragm.

Of the 147 patients, 75 (51%) had postoperative MRI performed. Upon the evaluation of postoperative MRIs, RPD was present in 60 (80%) of 75 patients. Sixty-one (81%) of the 75 patients had an ELAPE performed. All 14 patients treated with c-APE had RPD in both posterior quadrants of the pelvis on postoperative MRI (Figure 4). Thus, in the included c-APEs, the muscular pelvic diaphragm was retained as intended.

**Figure 5:**
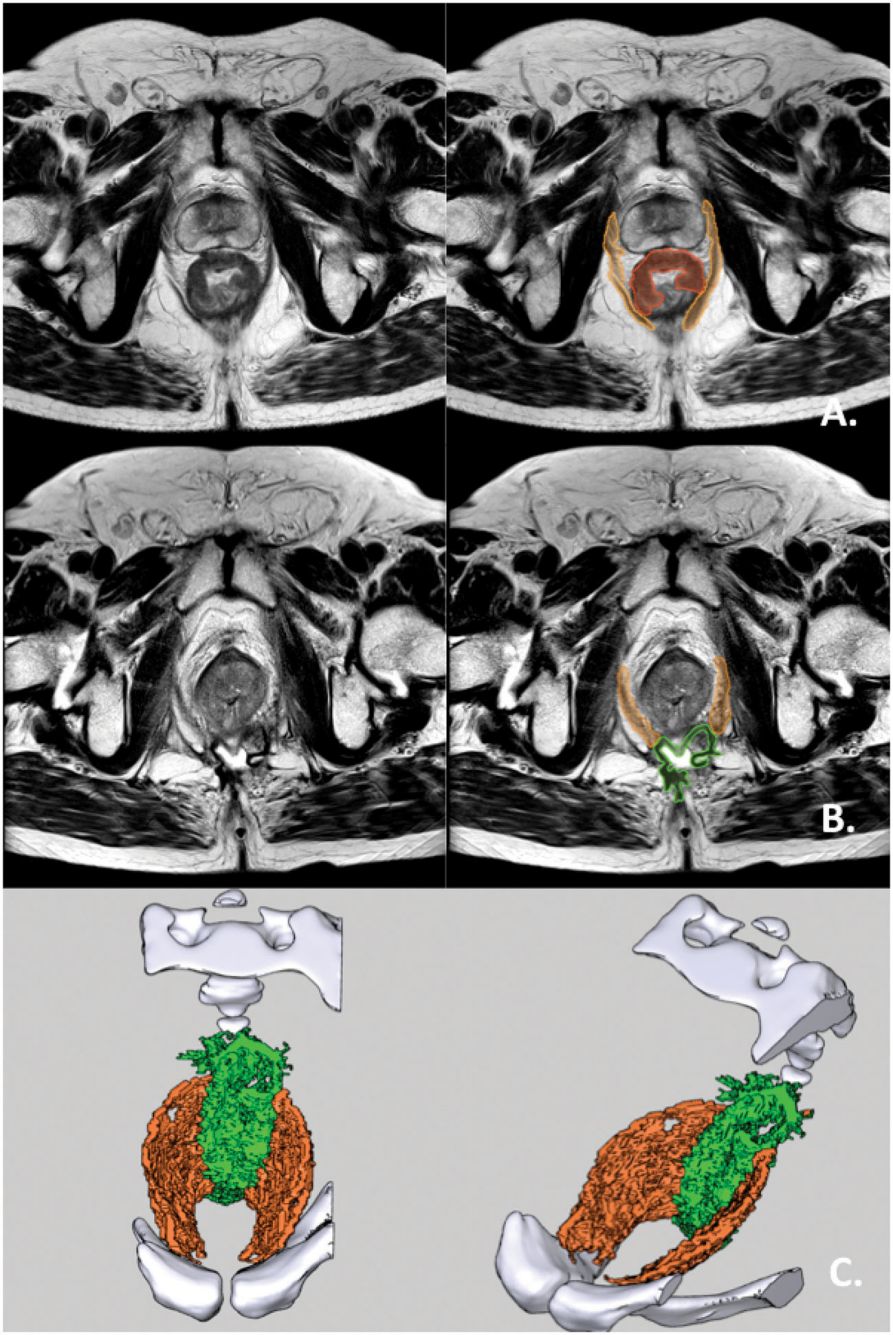
ELAPE with RPD in both posterior quadrants. **(A)** Preoperative axial T2-weighted MRI of a rectal cancer patient with an mrT3 tumor with invasive growth from 9 o'clock to 2 o'clock. The pelvic diaphragm is hatched in orange. The tumor is hatched in red. **(B)** Postoperative axial T2-weighted MRI of the same patient. RPD hatched in orange while supporting mesh hatched in green. **(C)** The pelvic diaphragm of the same patient, 3D-rendered and shown in superior and antero-lateral superior views. The rendering is made from the above postoperative MRI. The bony pelvis is rendered in white. RPD is rendered in orange. Supporting mesh is rendered in bright green.

**Figure 6:**
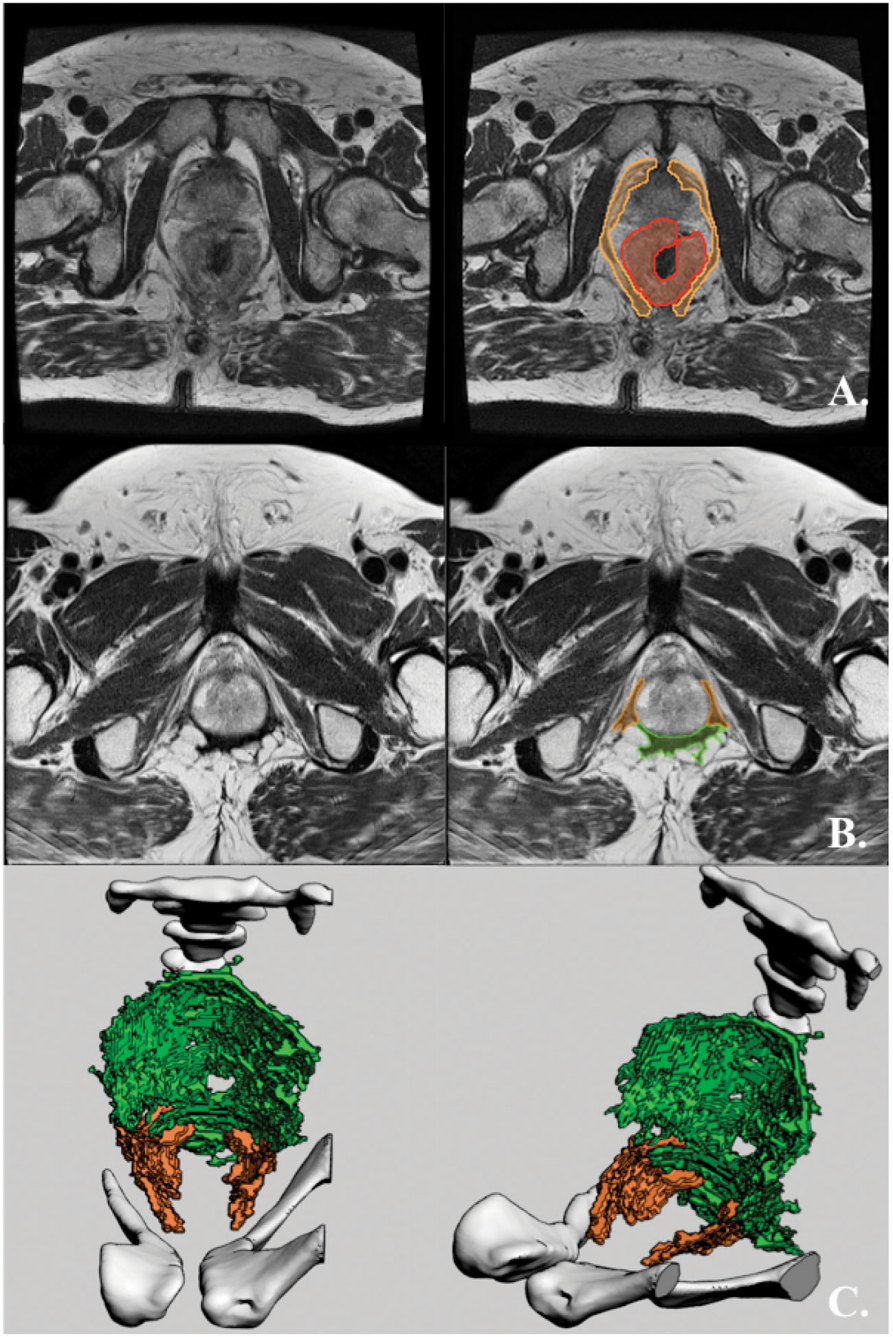
ELAPE without RPD in the posterior quadrants. **(A)** Preoperative axial T2-weighted MRI of a rectal cancer patient with an mrT3 tumor with circumferential growth (from 1 o'clock to 1 o'clock) and invasive growth from 3 o'clock to 8 o'clock. RPD is hatched in orange, while the tumor is hatched in red. **(B)** Postoperative axial T2-weighted MRI of the same patient. RPD hatched in orange while supporting mesh hatched in green. **(C)** The pelvic diaphragm of the same patient, 3D-rendered and shown in superior and antero-lateral superior views. The rendering is made from the above postoperative MRI. The bony pelvis is rendered in white, RPD is rendered in orange, and supporting mesh is rendered in bright green.

The residual pelvic diaphragm in any posterior quadrant of the pelvis was identified on postoperative MRI in 46 of 61 ELAPEs (75%, Table 2). Of 46, 13 had RPD in one posterior quadrant (3-6 o'clock OR 6-9 o'clock, as per Figure 3), while 33 had RPD in both posterior quadrants (Figure 5). The remaining 15 had no visible RPD in either posterior quadrant (Figure 6). Although performed with the intent to completely excise the pelvic diaphragm in the posterior quadrants, this was not achieved in 75% of ELAPEs.

**Table 2:** RPD on postoperative MRI, ELAPE-subgroup.

	***N* = 61**	**Residual pelvic diaphragm** **(*n* = 46)**	**No residual pelvic diaphragm** **(*n* = 15)**	***p* value**
Sex ratio (M:F)		29:17	8:7	0.504
Distance of primary tumor to anal verge by rigid proctoscopy (cm)^€^				0.455
→ 0–1.9	4	2 (50)	2 (50)	
→ 2–3.9	27	22 (81)	5 (19)	
→ 4–5.9	17	12 (70)	5 (30)	
→ >6	4	3 (75)	1 (25)	
→ Missing	9	7 (78)	2 (22)	
Neoadjuvant therapy				0.061
→ None	28	18 (64)	10 (36)	
→ CRT	33	28 (85)	5 (15)	
CRM^$^				>0.999
→ Involved	7	5 (71)	2 (29)	
→ Not Involved	54	41 (76)	13 (24)	
Tumor location on MRI				0.266
→ Anterior	29	20 (69)	9 (31)	
→ Other	32	26 (81)	6 (19)	
Pathological T-category^§,€^				0.194
→ pT0	5	4 (80)	1 (20)	
→ pT1	5	5 (100)	0 (0)	
→ pT2	23	16 (70)	7 (30)	
→ pT3	25	20 (80)	5 (20)	
→ pT4	3	1 (33)	2 (67)	
Mesorectal plane of surgery^€^				0.867
→ Mesorectal	17	13 (76)	4 (24)	
→ Intramesorectal	16	13 (81)	3 (19)	
→ Musc. Propria	28	20 (71)	8 (29)	
Perineal plane of surgery				0.981
→ Extralevator plane	8	6 (75)	2 (25)	
→ Sphincteric plane	27	21 (78)	6 (22)	
→ Intramuscular/ submucosal plane	25	19 (76)	6 (24)	
→ Not reported	1	0 (0)	1 (100)	
Local Recurrence				>0.999
→ Yes	6	5 (83)	1 (17)	
→ No	55	41 (75)	14 (25)	

*Values in parentheses are percentages unless indicated otherwise*.

*Residual levator was defined as any muscular pelvic diaphragm visible on MRI in the two posterior quadrants of the pelvis, i.e., between 3 and 9 o'clock*.

§ *Based on pathological evaluation of excised specimen (the pathological tumor category for the 33 patients who had preoperative adjuvant therapy (ypT) was: T0, 5; T1, 4; T2, 9; T3, 13; T4, 2)*.

$ *Fischer's exact test*.

€ *Freeman–Halton test*.

*CRM, circumferential resection margin; MRI, magnetic resonance imaging*.

In those treated with ELAPE, male sex, neoadjuvant therapy, the distance of primary tumor to the anal verge, involved CRM, tumor orientation, pathological T-category, surgical planes, and local recurrence were not found to be univariate risk factors of RPD (Table 2).

### Circumferential Resection Margin Involvement

Twenty-three (16%) of 147 patients had an involved CRM at pathological evaluation. An increased risk of involved CRM after surgery was observed in anteriorly oriented tumors with 16 (22%) of 73 after surgery compared with 7 (9%) of 74 non-anteriorly oriented tumors (*p* = 0.038). The increased risk in anteriorly oriented tumors was the same in the subgroup of patients treated with ELAPE (*n* = 125, *p* = 0.038). Advanced pathological tumor stage (*p* < 0.001), venous invasion (*p* < 0.001), regional lymph node involvement (*p* < 0.001), and tumor height above 6 cm by rigid proctoscopy (p= 0.034) were also found to be univariate risk factors for an involved CRM (Table 3).

**Table 3:** Full cohort by CRM (*n* = 147).

	**CRM+** **(*n* = 23)**	**CRM–** **(*n* = 124)**	***p* value**
Sex ratio (M:F)	11:12	80:44	
Age (years)^*^	68	67	
Distance of primary tumor to anal verge by rigid proctoscopy (cm)			0.034
→ 0–1.9	1 (10)	9 (90)	
→ 2–3.9	9 (17)	45 (83)	
→ 4–5.9	4 (9)	38 (91)	
→ ≥6	5 (42)	7 (58)	
→ Missing	4 (14)	25 (86)	
Neoadjuvant therapy			0.655
→ None	7 (13)	47 (87)	
→ CRT	16 (17)	77 (83)	
T-category on MRI^€^			0.656
→ T2	4 (11)	32 (89)	
→ T3	11 (14)	67 (86)	
→ T4	8 (25)	24 (75)	
→ Tx	0 (0)	1 (100)	
Tumor orientation on MRI			0.038
→ Anterior	16 (22)	57 (78)	
→ Other	7 (9)	67 (91)	
Surgery^$^			>0.999
→ ELAPE	20 (16)	105 (84)	
→ c-APE	3 (14)	19 (86)	
Pathological T-category^§,€^			<0.001
→ pT0	0 (0)	10 (100)	
→ pT1	1 (20)	4 (80)	
→ pT2	0 (0)	48 (100)	
→ pT3	15 (21)	56 (79)	
→ pT4	7 (54)	6 (46)	
Venous invasion			<0.001
→ V0	7 (7)	99 (93)	
→ V1-V2	16 (39)	25 (61)	
Lymph node involvement			<0.001
→ N0	10 (9)	103 (91)	
→ N1-N2	13 (39)	20 (61)	
→ Missing	0 (0)	1 (100)	
Mesorectal plane of surgery			0.280
→ Mesorectal	3 (8)	36 (92)	
→ Intramesorectal	8 (19)	35 (81)	
→ Musc. Propria	12 (19)	52 (81)	
→ Not reported	0 (0)	1 (100)	
Perineal plane of surgery			0.128
→ Extralevator plane	1 (5)	18 (95)	
→ Sphincteric plane	8 (14)	51 (86)	
→ Intramuscular/submucosal plane	14 (23)	46 (77)	
→ Not reported	0 (0)	9 (100)	
Local recurrence			0.002
→ Yes	6 (55)	5 (45)	
→ No	17 (13)	119 (87)	

*Values in parentheses are percentages unless indicated otherwise*.

* *Values are median (range)*.

§ *Based on pathological evaluation of excised specimen (the pathological tumor category for the 94 patients who had preoperative adjuvant therapy (ypT) was: T0, 10; T1, 4; T2, 24; T3, 43; T4, 11)*.

$ *Fischer's exact test*.

€ *Freeman–Halton test*.

*CRM, circumferential resection margin; MRI, magnetic resonance imaging*.

### Correlation Between Histopathological Assessment and Postoperative MRI

In 24 (32%) of the 75 specimens, no pathological data were recorded on defects in the levator ani in the specimen. Of those remaining 51 specimens (45 ELAPE, 6 c-APE), the pathologist's re-evaluation based on standardized photographic documentation showed the presence of defects in the levator ani in 90% (46 of 51) and 89% (40 of 45) of those treated with ELAPE. In the subgroup of patients treated with ELAPE, findings of any RPD in posterior quadrants on postoperative MRI were in agreement with findings of any defects in the levator ani by pathological evaluation in 76% of cases (34 of 45).

### Local Recurrence

Local recurrence was detected in 11 (7%) of 147 patients within the follow-up period. Involved CRM was an independent risk factor for local recurrence (*p* = 0.002). Five (11%) of 46 patients with RPD after ELAPE developed local recurrence compared with 1 (7%) out of 15 of those who had no RPD.

## Discussion

During the study period, a standardized ELAPE was the procedure of choice, performed with the intent to remove all muscular pelvic diaphragms in the two posterior quadrants to reduce the risk of an involved margin. Inadvertent RPD was found in 46 (75%) of 61 postoperative MRIs of patients treated with ELAPE. Since RPD was detected in all patients who had a c-APE performed, we conclude that postoperative MRI of the pelvis reliably estimates the prevalence and localization of RPD.

An involved CRM was determined in 23 (16%) of 147 patients and associated with anteriorly located tumors.

A recent national Danish study has evaluated the rate of CRM positivity and surgical outcome after standard APE vs. ELAPE and found no difference in the outcomes following standard APE or ELAPE, but more patients suffered from wound complications and perineal pain after ELAPE (18, 21, 22). However, this was solely registry based and exact definitions of the surgical planes were lacking.

Anteriorly located tumors presented a univariate risk factor for CRM involvement (22%). Among those treated with ELAPE, the comparatively low rate of involvement of the CRM in non-anteriorly oriented tumors (9%) in the present study suggests that patients with these tumors benefited from the wide posterior excision that is the hallmark of the ELAPE. The available literature emphasizes the importance of anterior dissection, as the CRM will be narrower in this area, particularly with anterior tumors (33). Preoperative evaluation and identification of patients at risk of positive CRM with MRI is crucial for appropriate tailoring of both neoadjuvant therapy and operative approach. This corresponds well with the notion that ELAPE would not reduce the risk of CRM involvement in anterior tumors compared with c-APE as the volume resected in the anterior compartment is essentially the same. Thus, choosing an ELAPE over a c-APE for an anteriorly oriented tumor provides no oncological benefit for the patient, although it retains its associated higher morbidity. Whether or not a tumor invades the anterior compartment should be carefully considered when deciding surgical approach. In these situations, a negative margin is feasible by extending the surgical plane into the anterior viscera for partial or en-bloc removal—individualizing the optimal surgical plane (34–36).

In mesorectal excision surgery, the mesorectal fascia presents an anatomical border, which may be readily assessed for completeness of surgery—by MRI and pathological analysis alike. The attachment sites of the muscular pelvic diaphragm present no such solid anatomical border, and histopathology by definition only evaluates that which is removed. This leaves room for the pathologist over- or underestimating the amount of pelvic diaphragm left behind. Thus, in the case of ELAPE and c-APE, histopathological evaluation may be insufficient for the assessment of the completeness of surgery.

Low rectal cancer is a multifaceted malignancy that runs a highly variable course with a high risk of severe post-treatment outcomes. Algorithms for selecting a proper treatment course and measures for quality assurance should be multifaceted as well. Individualized surgery has been implemented in many leading surgical centers around the globe. Thus, unilateral ELAPEs and extended c-APEs (c-APE with a slightly wider resection of the pelvic diaphragm without reaching sites of attachment to the pelvis) are often performed today. This has become possible due to a better understanding of the individual case, which may be preoperatively visualized by MRI and discussed at a preoperative MDT conference. As technical advancements continually improve outcomes for patients with low rectal cancer, postoperative MRI may be used for quality control of intended surgery. The authors recommend individualized surgery implemented in a standardized program that includes quality control by MRI after surgery, thus eliminating the use of and reliance on self-reported classifications.

This study enjoys a large degree of data completeness at the individual patient level. Data were collected prospectively for all included patients. As the initial cohort contained all patients with low rectal cancers treated with either ELAPE or c-APE at our treatment center, the patients constitute a consecutive, unselected cohort. The same MRI protocol was adhered to for all patients. None of the previous studies on the subject of ELAPE contained an in-depth analysis of circumferential tumor orientation, although Battersby et al. and Salerno et al. previously described anterior tumor location on MRI as a risk factor for the involvement of CRM in low rectal cancer (37, 38). No previous studies on ELAPE have used postoperative MRI for the assessment of surgical planes. Unfortunately, the data were not stratified by the individual surgeon, which would have enabled us to account for possible operator-dependent differences in outcomes.

In conclusion, RPD after any APE can be depicted by postoperative MRI and was found in the posterior quadrants of the pelvis in 46 (75%) of 61 patients treated with ELAPE. Anterior tumor orientation was a risk factor for CRM involvement regardless of chosen surgical technique.

## Data Availability Statement

The raw data supporting the conclusions of this study will be made available by the authors upon request, without undue reservation.

## Ethics Statement

In 2007, an audit on quality of rectal cancer treatment and surgery was implemented at Aarhus University Hospital, Denmark. The audit was part of a large regional audit with focus on postgraduate training of colorectal MDTs in North and Central Denmark Regions. This study was approved as a quality assurance project with no need for oral or written consent required by Danish law.

## Author Contributions

KMO: main author and researcher. PB: project co-supervisor. SL: senior advisor. RH-M: pathological evaluations and expertise. HC: contribution of surgical expertise. HL: contribution of expertise in 3D rendering of MRIs. BGP: main project supervisor.

## Funding

This study was funded by the Danish Cancer Society, Aarhus University Hospital, Aarhus University, and Eva and Henry Frænkel's Memorial Fund.

## Conflict of Interest

The authors declare that the research was conducted in the absence of any commercial or financial relationships that could be construed as a potential conflict of interest.

## Publisher's Note

All claims expressed in this article are solely those of the authors and do not necessarily represent those of their affiliated organizations, or those of the publisher, the editors and the reviewers. Any product that may be evaluated in this article, or claim that may be made by its manufacturer, is not guaranteed or endorsed by the publisher.
